# Relationship between body mass, lean mass, fat mass, and limb bone cross‐sectional geometry: Implications for estimating body mass and physique from the skeleton

**DOI:** 10.1002/ajpa.23398

**Published:** 2018-01-18

**Authors:** Emma Pomeroy, Alison Macintosh, Jonathan C.K. Wells, Tim J. Cole, Jay T. Stock

**Affiliations:** ^1^ School of Natural Sciences and Psychology Liverpool John Moores University Liverpool, L3 3AF United Kingdom; ^2^ ADaPt Project, PAVE Research Group, Department of Archaeology and Anthropology University of Cambridge Cambridge, CB2 3QG United Kingdom; ^3^ UCL Great Ormond Street Institute of Child Health London, WC1N 1EH United Kingdom; ^4^ Department of Anthropology University of Western Ontario London Ontario, N6A 3K7 Canada

**Keywords:** fat mass, human evolution, lean mass, osteology

## Abstract

**Objectives:**

Estimating body mass from skeletal dimensions is widely practiced, but methods for estimating its components (lean and fat mass) are poorly developed. The ability to estimate these characteristics would offer new insights into the evolution of body composition and its variation relative to past and present health. This study investigates the potential of long bone cross‐sectional properties as predictors of body, lean, and fat mass.

**Materials and Methods:**

Humerus, femur and tibia midshaft cross‐sectional properties were measured by peripheral quantitative computed tomography in sample of young adult women (*n* = 105) characterized by a range of activity levels. Body composition was estimated from bioimpedance analysis.

**Results:**

Lean mass correlated most strongly with both upper and lower limb bone properties (r values up to 0.74), while fat mass showed weak correlations (*r* ≤ 0.29). Estimation equations generated from tibial midshaft properties indicated that lean mass could be estimated relatively reliably, with some improvement using logged data and including bone length in the models (minimum standard error of estimate = 8.9%). Body mass prediction was less reliable and fat mass only poorly predicted (standard errors of estimate ≥11.9% and >33%, respectively).

**Discussion:**

Lean mass can be predicted more reliably than body mass from limb bone cross‐sectional properties. The results highlight the potential for studying evolutionary trends in lean mass from skeletal remains, and have implications for understanding the relationship between bone morphology and body mass or composition.

## 1 INTRODUCTION

Body mass can be divided into two major components: body fat (energy stores) and lean mass (including muscle, organs, and bone), each of which has distinct biological significance and was likely subject to different selective pressures during human evolution. Humans have a high proportion of body fat compared to other primates, and to mammals more widely (Pontzer et al., 2016; Wells, 2010; Zihlman & Bolter, 2015). In contrast, skeletal muscle mass (a major constituent of lean mass) is low compared with our closest relatives *Pan* (Zihlman & Bolter, 2015), other primates (Muchlinski, Snodgrass, & Terranova, 2012) and, it has been argued, earlier fossil hominin species (Churchill, 1998; Churchill, 2006; Trinkaus, 1983; Trinkaus et al., 1991; Wells, 2017). Within our species, fat and lean masses vary in relation to selective pressures such as climate and disease load (Houghton, 1990; Wells, 2012a, 2012b; Wells & Cortina‐Borja, 2013; Wilberfoss, 2012), and population variation in body composition is linked to contemporary disease susceptibility (Gysel et al., 2014; Lear, Kohli, Bondy, Tchernof, & Sniderman, 2009; Unni et al., 2009; Wells, 2016). The ability to estimate fat and lean mass from skeletal characteristics would offer novel potential to investigate past human adaptation, health and evolution, as well as to understand the origins of contemporary variation in body composition.

Typically, body mass is estimated from the skeleton from femoral head diameter (Grine, Jungers, Tobias, & Pearson, 1995; McHenry, 1992; Ruff, Scott, & Liu, 1991; Ruff, Trinkaus, & Holliday, 1997), from bi‐iliac breadth and stature (Auerbach & Ruff, 2004; Ruff, 2000a; Ruff et al., 1997; Ruff, Niskanen, Junno, & Jamison, 2005; Schaffer, 2016: see Auerbach and Ruff, 2004, for a review), or less commonly from other joint and shaft dimensions or properties (Aiello & Wood, 1994; De Groote & Humphrey, 2011; Elliott, Kurki, Weston, & Collard, 2016a,b; Grabowski, Hatala, Jungers, and Richmond, 2015; Grine et al., 1995; Lorkiewicz‐Muszyńska et al., 2013; McHenry, 1992; Moore, 2008; Moore and Schaefer, 2011; Ruff 2007; Ruff et al., 1997; Squyres and Ruff, 2015; Wheatley, 2005; Will and Stock, 2015). While the estimation of body mass from the skeleton is relatively routine in osteology, despite its known inaccuracy (Elliott et al., 2016a; Heyes & MacDonald, 2015), fewer studies have explored methods for estimating body mass components. Previous attempts have largely focused on estimating muscle area in relation to bone cross‐sectional properties at one body location (e.g. forearm), rather than total skeletal muscle or lean mass, and have produced mixed results. Shaw (2010) reported that bone cross‐sectional geometry was a relatively poor predictor of muscle area at the same cross‐sectional location for the humerus, ulna, and tibia of adult male athletes residing in the United Kingdom, although he reported correlations of up to 0.57 for the humerus, despite adjusting models for body mass (which may have removed a significant portion of any relationship). Slizewski, Schönau, Shaw, and Harvati (2013) and Slizewski, Burger‐Heinrich, Francken, Wahl, and Harvati (2014) reported stronger results for the ulna among a German sample of mixed sex and age.

The problem of estimating whole body lean mass and fat mass has received less attention. The theoretical basis of “mechanical” methods of estimating body mass is that joints, particularly of the lower limb in humans, are adapted to, and so are proportional in size to, the load they support (Auerbach & Ruff, 2004). By the same rationale, cross‐sectional geometry of the major limb bones is known to respond to mechanical loading (e.g., Bass et al., 2002; Frost, 1988, 2003; Haapasalo et al., 2000; Pearson and Lieberman, 2004; Ruff, Holt, and Trinkaus, 2006; Shaw, 2008; Shaw and Stock, 2009; Stock and Pfeiffer, 2001), and so could also be used to estimate body mass and its components, although this is not widely practiced (but see, e.g., Robbins, Sciulli, and Blatt, 2010 with juveniles). While activity levels influence bone cross‐sectional geometry (Ruff, 2008; Ruff, Trinkaus, Walker, & Larsen, 1993), body mass accounts for 80% of the variation in cross‐sectional geometry (Davies, 2012). Interestingly, some studies suggest that joint size and cross‐sectional shaft geometry are more closely related to lean mass than to body mass (Reeves, 2014; Ruff et al., 1991; Semanick et al., 2005; Wu et al., 2007), although this has not been extensively investigated.

As components of overall mass and bone loading, both total lean and total fat masses (hereafter lean and fat masses) may individually relate to joint sizes and cross‐sectional bone properties. However, the influence of muscle forces on bone loading appears to be much greater than that of gravity and body mass per se (Baker et al., 2013; Beck et al., 2001a; Burr, 1997; Capozza, Cointry, Cure‐Ramírez, Ferretti, & Cure‐Cure, 2004; Hsu et al., 2006; Petit et al., 2005; Robling, 2009). Bone and skeletal muscle are proposed to form a “functional unit” so that bone cross‐sectional properties respond to muscle mass and strength to maintain mechanical integrity (Edwards et al., 2013; Fricke & Schoenau, 2007; Judex, Zhang, Donahue, & Ozcivici, 2016; Parfitt, 1997; Puthucheary et al., 2015; Rauch & Schoenau, 2001; Schoenau, 2005; Schoenau & Fricke, 2006: but see, e.g., Judex et al., 2016) through a feedback mechanism (Frost, 1988, 1997, 2003). As bone and skeletal muscle derive from common progenitor cells from the somatic mesoderm and achieve peak tissue mass at the same time, they may also show correlated properties resulting from common genetic and environmental influences during development (DiGirolamo, Kiel, & Esser, 2013; Karasik et al., 2009; Lang et al., 2009; Mikkola et al., 2009; Seeman et al., 1996). Work by Ruff (2003) suggests that the relative importance of gravitational and muscular forces varies by limb, the former being more important for the lower limb and the latter for the upper limb, particularly in males. Adjusting for body mass, there was a strong correlation (*r* = 0.70) between the residuals of muscle area and humeral shaft strength in the oldest individuals (17 years) in the same dataset (Ruff, Burgess, Ketcham, & Kappelman, 2016).

The theoretical basis for a link between fat mass and bone properties is weaker. Both bone shaft size and mechanical properties are more closely related to lean mass than to fat mass, and fat mass is not a strong predictor of bone size or geometry (Bailey & Brooke‐Wavell, 2010; Beck et al., 2001a, 2009; Cole et al., 2012; El Hage & Baddoura, 2012; Farr et al., 2014; Hu et al., 2012; Leslie et al., 2014; Mallinson, Williams, Hill, & De Souza, 2013; Moon et al., 2015; Semanick et al., 2005; Sioen, Lust, De Henauw, Moreno, & Jiménez‐Pavón, 2016; Taes et al., 2009; Travison, Araujo, Esche, Beck, & McKinlay, 2008; Wu et al., 2007). Most of these studies focused on femoral neck geometry inferred from dual energy X‐ray absorptiometry (DXA), but peripheral quantitative computed tomography (pQCT) studies of the tibia (Baker et al., 2013; LeBrasseur, Achenbach, Melton, Amin, & Khosla, 2012; Taes et al., 2009) and radius (LeBrasseur et al., 2012; Taes et al., 2009) report similar results. However, there are several grounds on which we might predict a relationship between limb bone cross‐sectional properties and adiposity: fat mass is a component of body mass and therefore contributes to skeletal loading; Bone medullary adipose tissue (BMAT) may show an inverse relationship with body mass and shares common progenitor cells with osteoblasts (reviewed in Devlin, 2011; Devlin and Rosen, 2015; Fazeli et al., 2013; Scheller, Cawthorn, Burr, Horowitz, and MacDougald, 2016; Scheller and Rosen, 2014); and bone is a source of hormones that contribute to the regulation of energy balance (Zhang, Riddle, & Clemens, 2015).

The purpose of this study is to examine the relationships between long bone cross‐sectional properties, body mass, and estimates of lean, muscle, and fat mass using a sample of young adult women of varying activity levels, and known body mass and composition. The aim is to test the feasibility of estimating body mass and its components from long bone shaft properties, independently of stature. Based on previous studies we hypothesize that lean mass will show the closest relationships to bone cross‐sectional properties, followed by body mass, with fat mass showing the weakest correlations. It has previously been argued that bone properties of the lower limb should more closely relate to body mass (and by extension its components) in humans since the upper limb does not routinely support body mass beyond infancy (Ruff, 2003; Ruff, Trinkaus, Walker, & Larsen, 1993; Schoenau, Neu, Mokov, Wassmer, & Manz, 2000; Slizewski et al., 2013; Trinkaus & Churchill, 1999). Therefore we also predict that bones of the lower limb (tibia, femur) will have stronger relationships to body mass and its components than those of the upper limb (humerus).

## 2 MATERIALS AND METHODS

### 2.1 Study sample

The sample consists of 105 healthy women aged between 19 and 40 years, with no history of medical conditions or medication use known to interfere with bone metabolism. The largest portion of the sample (97 women) was recruited via a study of musculoskeletal adaptation to behavior as part of the ADaPt Project, University of Cambridge, UK. Participants included varsity level rowers, soccer players, and endurance runners recruited from the Cambridge University Women's Boat Club, Women's Association Football Club, Athletics Club, Hare & Hounds, and Triathlon Club, as well as the Cambridge & Coleridge Athletics Club, and the Cambridge Triathlon Club. Recreationally‐active controls were recruited through several University of Cambridge colleges and the University of Cambridge Graduate Union. An additional eight participants were recruited via a study of ultramarathon runners as part of the ADaPt Project, from the Beyond the Ultimate Jungle Ultra 2016 and Everest Trail Race 2016. Both studies were approved by the Cambridge University Human Biology Research Ethics Board (HBREC.2015.25 and HBREC.2016.14) and ethical approval for the use of peripheral quantitative computed tomography (pQCT) was obtained from the NHS Health Research Authority NRES Committee East of England ‐ Cambridge East (15/EE/0017). All volunteers provided prior written informed consent.

The dataset is particularly suited to investigating relationships between bone properties and body mass and its components, since it includes women engaged in a wide range of physical activity levels, and sports which impose a variety of loading regimes on the upper and/or lower body. Given that people are thought to have been more active in the past, particularly prior to the Holocene (Ruff et al., 2015, 1993; Ryan & Shaw, 2015; Shaw, 2010: but see Pontzer et al. 2012), this sample is more likely to encompass a range of variation in musculature and activity levels that will parallel both past and modern loading regimes on the skeleton, making the results of our analyses more relevant for both contemporary and past populations. As only women are included in the dataset, the aim is not to create a full set of regression equations that can be applied, but to test of the feasibility of such an approach.

### 2.2 Anthropometry

Stature was measured to the nearest mm using a SECA 274 stadiometer, and body mass was measured to the nearest 0.1 kg with the participant dressed in light athletic clothing using a SECA electronic scale. Humerus, femur, and tibia lengths were measured following International Standards for Anthropometric Assessment (2001), using sliding callipers to the nearest 0.1 cm. It should be noted that femur length was measured from the superior border of the greater trochanter to the distal‐most part of the lateral condyle, and so is not directly equivalent to the maximum or bicondylar femur lengths typically used in osteology.

### 2.3 Estimation of body composition

Lean mass (muscle, organ, and bone weight) and fat mass were estimated by bioimpedance analysis (BIA) using a Bodystat QuadScan 4000 (Bodystat, Isle of Man, UK). Briefly, BIA passes a current through the body between electrodes placed on the hands and feet with the participant supine, and an estimate of total body water is obtained by measuring resistance and reactance to the current and adjusting them for height. Total body water is then converted to estimates of fat and lean mass using age‐ and sex‐specific equations built into the equipment.

### 2.4 Bone properties

**Figure 1:**
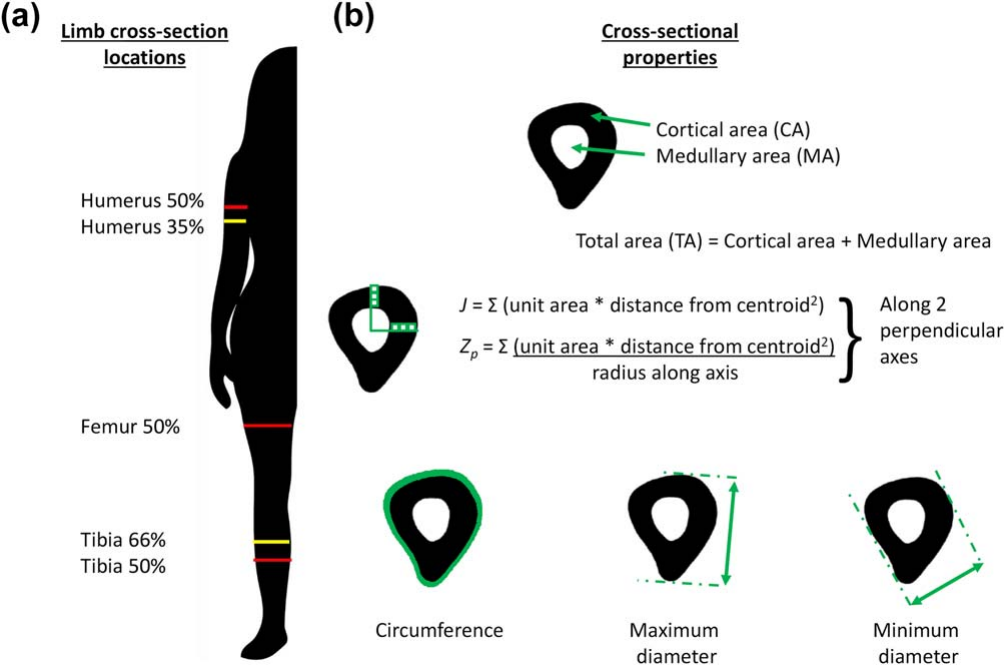
Bone cross‐section locations (A) and cross‐sectional properties (B) used in this study. Cross‐section illustrated is femur 50%. Results are reported in detail for the humerus, femur and tibia midshaft (50%) locations (red)

Peripheral Quantitative Computed Tomography was performed on both humeri (35% and 50% of length, measured from the distal end), and the right femur (at 50% of length), and tibia (at 66% and 50% of length: Figure 1A) using a Stratec XCT‐3000 pQCT scanner (Stratec Medizintechnik GmbH, Pforzheim, Germany). Results are reported only for the right humerus, femur and tibia midshaft (50%) levels, as results from the 35% humerus and 66% tibia were similar to 50%, and those from the right humerus were very similar to those from the left. Images were visually screened, and any scans affected by movement artifacts were excluded; thus sample sizes vary slightly by measurement site.

Three classes of bone properties were investigated as predictors of body mass and its components (Figure 1B). First, the total (TA), cortical (CA), and medullary (MA) areas of each cross‐section in mm^2^ were analyzed, on the basis that a theoretical relationship has been predicted for total and cortical areas and body mass through skeletal loading, and between medullary cavity size and adiposity. Second, biomechanical properties representing bone strength (resistance to compressive forces) and rigidity (resistance to deformation) were included, again on the basis of theoretical relationships between loading, body mass and skeletal properties. Polar second moment of area (*J*, measured in mm^4^) represents torsional and twice average bending rigidity of the bone when modelled as a cylinder, and the polar section modulus (*Zp*, measured in mm^3^) represents torsional and twice average bending strength (Ruff, 2008). Finally, external dimensions of the bone cross‐section (maximum and minimum diameters and circumference) were included as these may be the only available data, for many older datasets or where cross‐sectional geometric analyses are not feasible. All bone properties were derived from the pQCT scans using the BoneJ plugin version 1.3.10 (Doube et al., 2010) for ImageJ version 1.46 (NIH: Rasband, 1997‐2016). Image stacks were thresholded using the “Optimise Threshold” function in BoneJ.

### 2.5 Data standardization

Stature is known to be an important predictor of lean body mass (e.g., Heymsfield, Gallagher, Mayer, Beetsch, & Pietrobelli, 2007; Heymsfield, Heo, Thomas, & Pietrobelli, 2011; Kulkarni et al., 2013), and any relationships between bone properties and lean mass could reflect overall size. Bone properties also relate to body size as previously outlined. Given that the relationship between stature and lean mass varies between populations, the ability to predict lean mass independently of stature would have distinct benefits for trying to investigate temporal or geographical variation in lean mass from skeletal remains. Furthermore, the intimate relationships between stature, body mass and its components, and bone properties, may mean that applying size adjustments to both variables may remove the relationship which would allow the prediction of body mass, lean mass or fat mass. Therefore this study investigates the relationships between lean mass and unstandardized bone properties. However, we separately adjust for stature to investigate to what extent bone properties relate to body mass, lean mass or fat mass as a result of overall body size.

### 2.6 Statistical analyses

Relationships between body mass, lean mass or fat mass, and bone properties were investigated using Pearson's correlation. Correlations were performed between body mass or its components and bone properties, as well as partial correlations adjusting for stature. Data were natural log transformed prior to correlation analysis as a number of the variables showed non‐normal distributions (determined by visual assessment of histograms and the ratio of skewness to its standard error), and to account for potential allometry.

Ordinary least squares (OLS) regression equations of body mass or its components on selected bone properties were fitted. One bone property from each type (area, cross‐sectional geometry and external measurements) from the tibial midshaft was used for trial regression models. Models were calculated with and without bone length, as an indicator of overall size, to see how it affected the model, and for raw and natural log transformed variables, to investigate whether potential allometry may result in a log‐log regression giving better results. The relative performance of the models was judged using the adjusted *R* ^2^ values and the Bayesian Information Criterion (BIC: Schwarz, 1978). The BIC offers an assessment of model fit, with lower values indicating better fit, which penalizes additional terms in the model to reduce the risk of over‐fitting. It is similar to the Akaike Information Criterion (AIC) but uses a larger penalty and hence leads to more parsimonious models. The summary statistics used to compare models here differ from those applied in some other studies, where mean prediction errors (PEs) and standard errors (SEEs: raw and as a percentage in both cases) are often quoted alongside *R* ^2^ values (e.g., Elliott, Kurki, Weston, and Collard, 2016b; Ruff et al., 2012; Squyres and Ruff, 2015). However, where log‐log regression models are used (e.g., Elliott et al., 2016b), these measures are not appropriate. Working on the natural log scale is effectively working in percentage terms (Cole, 2000; Cole & Altman, 2017), and thus calculating further percentages (%SEE, %PE) is inappropriate. The SEE of the log‐log regression model is directly interpretable in percentage units. Therefore 100 x SEE of the log–log regression models and %SEE (100 × (SEE/Mean y)) of the raw models are presented for comparison with each other and with other published models.

All analyses were conducted using SPSS for Windows v. 24.0 (IBM Corporation, Chicago), with *p* values < 0.05 considered significant.

## 3 RESULTS

Demographic information and summary statistics on the study sample is presented in Table 1, and by individual sports disciplines and for controls in Supporting Information Table 1. Mean age was 24 years, one third of the sample were relatively sedentary controls, 38% were rowers and the remainder were endurance or ultramarathon runners, soccer players or ex‐athletes. The vast majority (97%) were of European ancestry, 71% reported using some form of hormonal contraceptive in the past, and 45% reported current hormonal contraceptive use. Percentage body fat was 25% for the controls and 21% for the athletes.

**Table 1:** Characteristics of the study sample

	Control (*n* = 34)		Athlete (*n* = 71)		Total (*n* = 105)	
Variable	Mean	Standard Deviation	Mean	Standard Deviation	Mean	Standard Deviation
Age (years)	23	3	24	6	24	5
Stature (cm)	167.9	7.4	170.5	7.6	169.7	7.6
Body mass (kg)	61.7	11.1	65.1	9.5	64.0	10.1
BMI (kg/m^2^)	21.9	3.9	22.3	2.4	22.2	3.0
Lean mass (kg)^a^	45.6	5.8	51.1	6.7	49.3	6.9
Fat mass (kg)	16.0	6.9	13.9	4.4	14.6	5.4
Percent fat mass (%)^a^	25.2	6.4	21.1	4.9	22.4	5.7

Athletes comprised 40 rowers, 11 endurance runners, 8 ultramarathon runners, 11 soccer players, and 1 ex‐athlete (gymnast).

a Significant difference between athletes and controls, *p* < 0.001 by independent samples T test. All other comparisons not significant.

**Figure 2:**
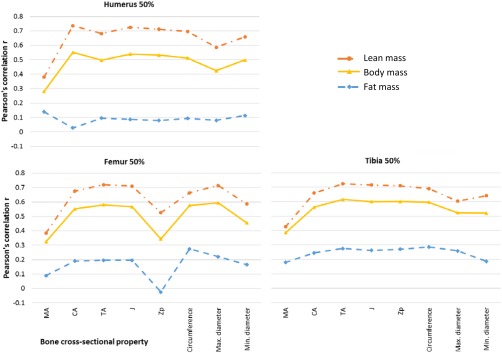
Correlations between body mass, lean mass or fat mass and bone properties. TA = total area; CA = cortical area; MA = medullary area; *J* = polar second moment of area; *Zp* = polar section modulus

Correlations between log‐transformed variables are summarized in Table 2 and Figure 2. The highest correlations for each tissue component were as follows: body mass, tibia midshaft TA (*r* = 0.62); lean mass, humerus midshaft CA (*r* = 0.74); and fat mass, tibia midshaft circumference (*r* = 0.29). For all bone properties at all cross‐section locations, correlations were lowest for fat mass, highest for lean mass, and intermediate for body mass. Generally, the pattern of strength of correlations was similar for body mass, lean mass, and fat mass across the different bones and cross‐sections, except that medullary area had the lowest correlations with lean mass and body mass, but highest correlations with fat mass. The strongest correlations with lean and body mass were generally CA, *J*, and *Zp*. External bone measurements generally had weaker correlations, although of those, circumference was generally strongest. Correlations between bone properties and fat mass were relatively weak, but stronger for the lower than the upper limb.

**Table 2:** Correlations between body mass, lean mass, or fat mass and bone properties (all variables log transformed)

	Unadjusted			Adjusted for stature		
	Body mass	Lean mass	Fat mass	Body mass	Lean mass	Fat mass
**Humerus 50%**						
TA (mm^2^)	0.50	0.68	0.10^a^	0.25	0.55	0.01
CA (mm^2^)	0.55	0.74	0.03^a^	0.23	0.60	−0.14
MA (mm^2^)	0.28	0.38	0.14^a^	0.15	0.16	0.15
*J* (mm^4^)	0.54	0.73	0.09^a^	0.26	0.59	−0.03
*Zp* (mm^3^)	0.53	0.71	0.08^a^	0.25	0.60	−0.06
Circumference (mm)	0.51	0.70	0.09^a^	0.24	0.53	0.02
Maximum diameter (mm)	0.42	0.59	0.08^a^	0.25	0.55	0.00
Minimum diameter (mm)	0.50	0.66	0.11^a^	0.28	0.47	0.11
**Femur 50%**						
TA (mm^2^)	0.58	0.72	0.20^a^	0.32	0.44	0.20
CA (mm^2^)	0.55	0.68	0.19^a^	0.34	0.48	0.18
MA (mm^2^)	0.33	0.38	0.09^a^	0.05	0.01	0.06
*J* (mm^4^)	0.57	0.71	0.20^a^	0.31	0.44	0.20
*Zp* (mm^3^)	0.34	0.53	−0.02	−0.01	0.18	−0.08
Circumference (mm)	0.58	0.66	0.27	0.35	0.41	0.29
Maximum diameter (mm)	0.59	0.71	0.22	0.36	0.46	0.23
Minimum diameter (mm)	0.46	0.59	0.17	0.18	0.27	0.15
**Tibia 50%**						
TA (mm^2^)	0.62	0.73	0.28	0.40	0.51	0.29
CA (mm^2^)	0.56	0.66	0.25	0.38	0.49	0.24
MA (mm^2^)	0.39	0.43	0.18^a^	0.18	0.16	0.17
*J* (mm^4^)	0.60	0.72	0.26	0.39	0.52	0.27
*Zp* (mm^3^)	0.60	0.71	0.27	0.39	0.50	0.28
Circumference (mm)	0.60	0.69	0.29	0.39	0.48	0.30
Maximum diameter (mm)	0.52	0.60	0.26	0.31	0.35	0.26
Minimum diameter (mm)	0.52	0.64	0.19^a^	0.32	0.45	0.18

“a” denotes statistically non‐significant correlations (*p* > 0.05). TA = total area; CA = cortical area; MA = medullary area; *J* = polar second moment of area; *Zp* = polar section modulus.

**Table 3:** Adjusted R^2^ and Bayesian Information Criteria (BIC) for ordinary least squares regression models of tibia midshaft cross‐sectional properties for raw and natural log transformed variables

			BIC				Adjusted *R* ^2^				SEE			
			Basic model		Incl. bone length		Basic model		Incl. bone length		Basic model		Incl. bone length	
Dependent	Predictor	*n*	Raw	Log	Raw	Log	Raw	Log	Raw	Log	Raw	Log	Raw	Log
	TA	112	474.0	467.7	468.8	461.0	0.38	0.37	0.42	0.43	12.6	12.4	12.1	11.9
Body mass	*J*	112	477.8	471.1	470.5	462.2	0.36	0.36	0.42	0.42	12.8	12.6	12.2	11.9
	Circumference	112	479.0	471.9	471.5	462.9	0.35	0.35	0.41	0.42	12.9	12.7	12.3	12.0
	TA	104	333.6	331.9	317.5	316.0	0.52	0.52	0.60	0.60	9.7	10.0	8.9	8.9
Lean mass	*J*	104	338.4	334.7	319.1	315.5	0.50	0.51	0.60	0.60	10.0	10.0	8.9	8.9
	Circumference	104	344.3	331.9	324.4	322.0	0.47	0.48	0.58	0.58	10.3	10.2	9.2	9.1
	TA	104	352.9	322.0	357.5	326.4	0.06	0.07	0.05	0.06	36.1	33.1	36.3	33.2
Fat mass	*J*	104	353.3	322.8	358.0	327.1	0.06	0.06	0.05	0.05	36.2	33.2	36.4	33.3
	Circumference	104	352.3	321.4	356.9	325.8	0.07	0.07	0.06	0.07	36.0	33.0	36.2	33.1

TA = total area; *J* = polar second moments of area; Incl. bone length = model including bone length; SEE = standard error of estimate. Note that SEE column presents %SEE for raw data and SEE * 100 for log data. As described in the methods the natural log transformation results in SEEs which are already percentages (when multiplied by 100) and are thus comparable.

**Figure 3:**
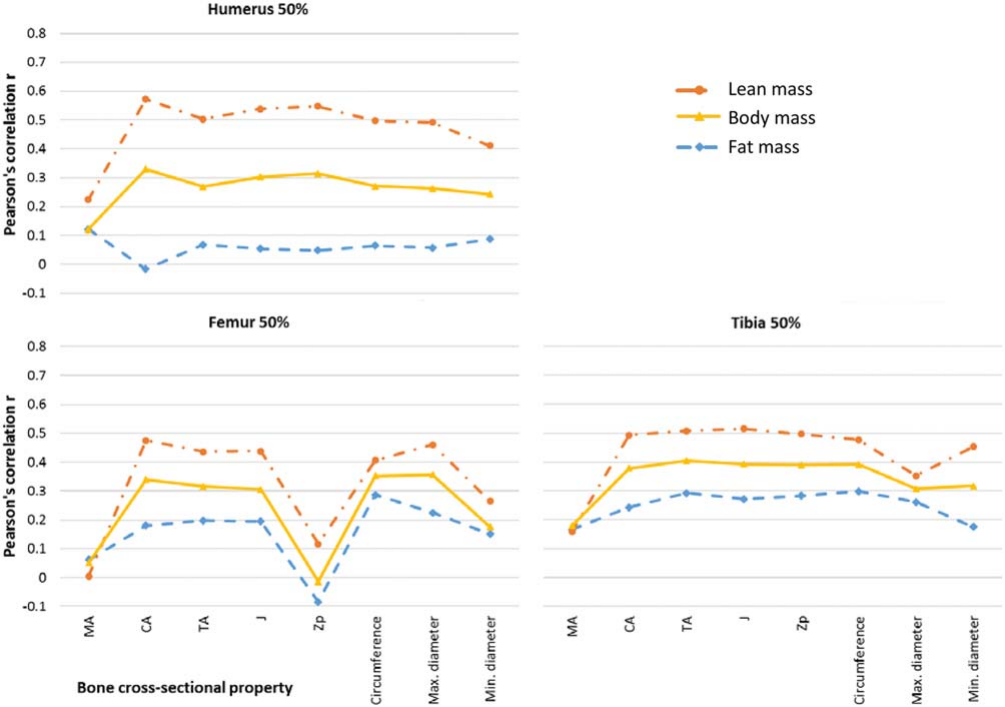
Partial correlations between body mass, lean mass or fat mass and bone properties, adjusting for stature. TA = total area; CA = cortical area; MA = medullary area; *J* = polar second moment of area; *Zp* = polar section modulus

Partial correlations adjusting for stature showed similar patterns for lean and body mass (Table 2, Figure 3) but correlations were typically 0.2 less showing that stature accounted for part, but not all, of the relationship between bone properties and lean or body masses. For fat mass, adjustment for stature had less impact, and as before fat mass was more closely related to lower than upper limb bone properties. The strongest correlations were between tibia midshaft TA for body mass (*r* = 0.40), humerus midshaft TA and *Zp* for lean mass (*r* = 0.60), and tibia midshaft circumference for fat mass (*r =* 0.30).

For the regression models (Table 3), *R* ^2^ values were highest for lean mass (0.47‐0.52), intermediate for body mass (0.35‐0.38), and low for fat mass (≤ 0.07). For all variables, the log‐log regression models gave lower BIC values, indicating that they fitted better than the untransformed models. Including bone length in the models increased *R* ^2^ values by 0.04‐0.07 for body mass, 0.20‐0.26 for muscle mass and 0.08‐0.11 for lean mass, and decreased BIC values. In contrast, *R* ^2^ values for fat mass remained essentially unchanged and adding bone length increased BIC. Thus the best models were those predicting lean mass using log‐transformed variables and including bone length.

## 4 DISCUSSION

This study demonstrates that in a sample of young adult women of varying habitual activity levels, the relationships between cross‐sectional properties of the humerus, femur and tibia on the one hand, and body mass and composition on the other, were strongest for lean mass, intermediate for body mass, and weakest for fat mass. OLS regression models derived for log‐transformed TA, *J* and circumference at the tibia midshaft had SEEs of 10% for lean mass and 12–13% for body mass, but only 33% for fat mass. These results for lean mass compare favorably with SEEs of 17.5% and 14.4% reported by Ruff et al. (1991) for body mass estimated from femoral head diameter and CA at the subtrochanteric level for white females. As indicated by those authors, the lack of remodelling in femoral head size coupled with weight gain between early late adolescence (when femoral head size is fixed) and body mass at the time of measurement may account for the weaker relationship between mass and femoral head size compared with shaft properties in their sample (Ruff et al. 1991), and compared with our relatively young and active adult female sample. The results for lean mass also compare reasonably well with SEEs of 6–8% for estimating body mass from bi‐iliac breadth and stature, using equations derived from population mean data (Ruff, 2000a).

Although previous studies have indicated a close relationship between stature and lean mass (e.g., Heymsfield et al., 2007, 2011; Kulkarni et al., 2013), the partial correlations demonstrate that stature explains some but not all lean mass variation. In the regression models using tibia midshaft properties, adding tibial length reduced the SEEs by 1–2% for lean mass. Bone length was added to the models, rather than stature, to maintain some independence between stature and estimated body mass or its components, and to avoid compound errors that would result from estimating stature from skeletal remains, and then including these estimates in the model for estimating body mass or its components. However, all long bone lengths show a relatively strong relationship to stature and so the inclusion of a bone length does not yield equations that would provide entirely stature‐independent estimates of body mass and its components.

It should also be noted that the femoral midshaft level used in this study (determined anthropometrically as half the distance between the greater trochanter and distal end of the lateral epicondyle) is not directly equivalent to the midshaft location that is typically derived from measurements on dry bone (i.e., 50% of maximum or bicondylar length). Thus any equations derived through the method we use for application to skeletal remains may need to be modified accordingly. Furthermore, given that stature is included in the equations used to estimate lean and fat masses from BIA, correlations between the variables may inflate their correlations with bone properties. However, the fact that correlations were only moderately attenuated when stature was controlled for suggests a genuine relationship between lean mass and bone properties.

These results are consistent with previous studies which suggested a stronger relationship between bone shaft cross‐section or joint surface properties and lean mass than with body mass (Reeves, 2014; Ruff et al., 1991; Semanick et al., 2005; Wu et al., 2007). Our findings support the argument that the relationship between bone and body mass is unlikely to be driven principally by the loading imparted by body mass due to gravity (Baker et al., 2013; Beck et al., 2001a; Burr, 1997; Capozza et al., 2004; Hsu et al., 2006; Petit et al., 2005; Robling, 2009).

The fact that correlations between bone properties and body composition were similar for the humerus as for the lower limb bones (femur and tibia) was unexpected. We considered the possibility that the high proportion of rowers in the sample (almost 40% of the total) could account for this result, but found this was not the case. Although much of the power in rowing comes from the legs, which experience forces over six times body weight, the arms also experience forces in excess of body weight (Hase et al., 2002). The higher loading on the arms experienced by rowers compared with other sportswomen and controls may mean that a higher proportion of lean mass is present in the arms in this sample, which might strengthen the relationship between humeral properties and lean mass among rowers, and so our sample as a whole. However, re‐running correlations between bone cross sectional properties excluding the rowers only slightly attenuated the relationships between humeral properties and lean mass, and actually had greater negative impact on the relationships between lower limb bone properties and lean mass (Supporting Information Table 2). This suggests that upper and lower limb bones are similarly related to total lean and body mass, with implications for understanding the relationships between lean mass and bone properties. Ruff (2003) reported that in a non‐adult longitudinal sample, the product of bone length and body mass was highly correlated with femoral strength and more weakly related to humeral strength, while humeral strength was more strongly correlated with muscle area among males, but the relationship was much weaker among females. The fact that our sample contains a majority of relatively muscular athletes may partially explain the difference from Ruff's (2003) results.

It has generally been assumed that in humans, as the lower limbs support body weight during locomotion after infancy whereas the upper limbs do not, a different relationship between body size, muscularity and bone cross‐sectional properties should apply for the upper and lower limbs (Ruff et al., 1993; Schoenau et al., 2000; Slizewski et al., 2013; Trinkaus & Churchill, 1999). Ruff (2000b) previously reported that cross‐sectional properties of upper and lower limb bones scaled similarly to body size, but noted that the correlations were stronger for lower limb bones than for those of the upper limb. This observation, along with our results, suggests that more systemic influences account for the relationship between whole body muscularity and bone cross‐sectional properties. Previous work indicates that increased loading in one area of the skeleton leads to bone deposition in other areas (Lieberman, 1996; Reeves, 2014). It has also been argued that common genetic influences on bone and skeletal muscle (DiGirolamo et al., 2013; Karasik et al., 2009; Lang et al., 2009; Mikkola et al., 2009; Seeman et al., 1996), as well as an intimate functional relationship between these tissues (the “muscle‐bone functional unit”), may explain relationships between muscle size (area, volume or mass) and bone size and mechanical properties including density and cross‐sectional geometry (Edwards et al., 2013; Fricke & Schoenau, 2007; H. Frost, 1988, 1997, 2003; Judex et al., 2016; Parfitt, 1997; Puthucheary et al., 2015; Rauch and Schoenau, 2001; Schoenau, 2005; Schoenau and Fricke, 2006: but see e.g., Judex et al., 2016), and our results are consistent with this interpretation.

The results do not support any close relationship between long bone shaft cross‐sectional properties and adiposity, similar to some previous studies (Beck et al., 2009; Petit et al., 2005; Travison et al., 2008; Wu et al., 2007), and indicate that estimating fat mass from skeletal properties would not be reliable. The relationship between body fat and bone appears complex, and while relationships between poor nutrition and increased marrow adipose tissue have been reported by a number of studies (reviewed in Devlin, 2011), these have not indicated whether this was accompanied by a change in bone architecture, particularly in the size of the medullary cavity as might be predicted. It is possible that such relationships can only be detected in a malnourished sample, and thus may not have been evident in a relatively well‐off and well‐nourished population such as that studied here. Alternatively, it may be that such alterations in the amount of BMAT are not reflected in the dimensions of the medullary cavity.

The dataset used in this study has some limitations. It is comprised of primarily young adult women, and was strongly dominated by women of European descent. The high proportion of physically active women and their selection primarily from among University students means that the sample is not representative of the adult female UK population. The relatively low body mass and BMI reflect this observation: the 2015 Health Survey for England reports a mean female BMI of 24.8 kg/m^2^ for age 16–24 years and 26.4 kg/m^2^ for age 24–35 years (Fuller, Mindell, & Prior, 2016), compared with 22.1 kg/m^2^ in our sample. For percentage body fat, the mean of 22% in our sample is substantially lower than that of 4,125 UK women reported by Flint, Cummins, and Sacker, (2014) at 36%. This may be the result of both the older mean age of Flint et al.'s sample (43 years) and the selection of athletes in our sample who are likely to be leaner than average women.

As it is likely that past populations were leaner than contemporary ones, our sample may be more appropriate than many contemporary samples selected from the general Western population for estimating body and lean mass in past populations. The prediction of body mass and its components may be more accurate for archaeological skeletons as the smaller proportion of body fat would give a closer relationship between bone properties and total mass. The use of modern Western (and thus more likely overweight) reference samples may lead to the overestimation of body mass in past individuals and populations who were leaner.

Furthermore, given known interpopulation variation in proportional skeletal muscle and lean mass, the extent to which ancestry might affect the relationship between bone cross‐sectional properties and lean mass needs to be explored. Baker et al. (2013) reported that greater tibial cross‐sectional area of “black” adults compared with “whites” was largely removed by adjustment for lean mass, suggesting that similar relationships between bone cross‐sectional properties and body mass components may exist across populations. Travison et al. (2008) reported a similar finding for proximal femoral strength among males, but further evaluation is needed.

The dataset was also based on BIA‐derived estimates of lean and fat mass. The “gold standard” method for measuring body composition is cadaver dissection, so clearly estimation techniques are the only option for living subjects (Wells & Fewtrell, 2006). While BIA is less accurate than magnetic resonance imaging (MRI), dual energy X‐Ray absorptiometry (DXA) or densitometry, the advantage is that BIA requires relatively simple equipment and causes minimal discomfort and inconvenience to subjects. Inaccuracies in the estimates of body mass components will of course attenuate the relationships between these characteristics and bone properties. Finally, the same analyses need to be repeated for men, given the known sex differences in body composition (Kirchengast, 2010; Wells, 2010), bone properties (Garn, Frisancho, Sandusky, & McCann, 1972; Lang, 2011; Schoenau et al., 2000) and hormonal influences on bone properties (Lapauw et al., 2009; Lorentzon, Swanson, Andersson, Mellström, & Ohlsson, 2005; Petit et al., 2004). Nonetheless, the data analyzed here serve to demonstrate that estimation of lean mass is promising and is likely to be more reliable than estimating body mass, and particularly fat mass, from cross‐sectional properties of the long bones.

A potential drawback of using cross‐sectional shaft properties is that they are known to be affected by age, sex, and activity levels (Ahlborg, Johnell, Turner, Rannevik, & Karlsson, 2003; Bass et al., 2002; Feik, Thomas, Bruns, & Clement, 2000; Frost, 1988, 2003; Garn, Rohmann, Wagner, & Ascoli, 1967; Haapasalo et al., 2000; Lazenby, 1990a, 1990b; Pearson & Lieberman, 2004; Ruff & Hayes, 1982; Ruff et al., 2006; Shaw, 2008; Shaw & Stock, 2009; Stock & Pfeiffer, 2001) and changes in body mass during life (Ruff et al., 1991). The relationship between bone cross‐sectional properties and activity may mean that to estimate body or lean mass from these properties, it would be most appropriate to use a reference sample of similar activity level. Apposition of bone to the periosteal surface and resorption of the endosteal surface progresses with age among adults (Ahlborg et al., 2003; Feik et al., 2000; Garn et al., 1967; Lazenby, 1990a, 1990b; Ruff and Hayes, 1982). Furthermore, muscle mass is known to decrease through adulthood in conjunction with bone density and geometry (Baker et al., 2013; Beck et al., 2001a; Mikkola et al., 2009), and changes in hormonal profiles, particularly the fall in estrogen associated with the menopause among women, are known to affect bone properties (Ahlborg et al., 2003; Beck et al., 2001b; Edwards et al., 2013; Melton III et al., 2000). This may have implications for estimating lean mass from the skeletons of individuals who were older at the time of death in studies of archaeological or paleoanthropological material.

There are two potential solutions, to derive equations from a sample with a wide age range so that age can be incorporated in the estimation equations, or to base predictions on bone properties that are unaffected by the ageing process. One such property might be joint size. We were unable to test associations between body mass, its components, and joint size using this dataset, but further investigation is warranted, given previous evidence that joint sizes are also more strongly related to lean mass than body mass (Reeves, 2014; Ruff et al., 1991; Semanick et al., 2005; Wu et al., 2007), and that they are minimally affected by age or activity due to functional constraints (Auerbach & Ruff, 2006; Buck, Stock, & Foley, 2010; Lazenby, Cooper, Angus, & Hallgrímsson, 2008; Lieberman, Devlin, & Pearson, 2001; Reeves, 2014; Ruff et al., 1991). Indeed the most appropriate type of bone property for estimating body mass may depend on the specific research questions posed. In some situations, it is desirable to know body or lean mass at the time of death (e.g., forensic cases, adjustment of bone biomechanical properties for loading due to body mass). In such cases, using cross‐sectional properties of the shaft, which are more plastic and responsive to changes in body mass, is likely to be more appropriate, providing a reference sample of similar activity levels is used.

On the other hand, to address other questions, such as examining trends in body size, health and growth in the past, it may be advantageous that noise introduced by life‐course changes in adult body mass is poorly captured by some skeletal measurements such as joint sizes. In essence, in these situations we are interested in what has been termed “basal body mass” in contemporary populations (Hruschka, Hadley, & Brewis, 2014), i.e., body mass in early adulthood before later accumulation of excess body fat due to ageing and lifestyle factors, or short term health variability. Such fluctuations in body mass are largely driven by changes in fat mass, which is especially plastic and sensitive to short term fluctuations in individual diet and health (Wells 2010), while lean mass appears to be less plastic and potentially subject to unique selective pressures (Hardikar et al. 2015; Houghton 1991; Prentice 2008; Steegmann 2007; Stini 1975; Wells et al. 2016; Wells 2012a; Wells and Shirley 2016; Wilberfoss 2012). As methods for estimating age at death from adult skeletons remain relatively imprecise (Buckberry 2015; Falys, Schutkowski, & Weston, 2006; Jackes 2000; Mays 2015) and age‐related aggregation of excess mass likely varies among populations, controlling for factors such as age‐related changes in body mass currently has limited potential. However, the fact that various studies indicate that skeletal dimensions best reflect body mass, and more precisely lean mass, in early adulthood drastically reduces the introduction of such noise into the data on early adult body size.

In conclusion, this study suggests that lean and body mass may be predicted relatively reliably from long bone cross‐sectional properties among adults. This could have multiple applications in studying changes in build and musculature in our evolutionary past, as well as in more recent populations. Our results demonstrate that this approach to estimating lean and body mass is worth pursuing further in larger, more diverse datasets in order to develop equations encompassing a wider range of age and ancestry and both sexes. Appropriate reference samples should be selected in terms of body mass and activity levels, as the use of relatively overweight modern Western reference samples may lead to the overestimation of body or lean mass based on skeletal properties. This is particularly the case where shaft cross‐sectional properties, known to be affected by age, activity and hormonal status, are employed.

## Supporting information

Additional Supporting Information may be found online in the supporting information tab for this article.

Supporting Information
