# Supplementary material for: Relationship between body mass, lean mass, fat mass, and limb bone cross‐sectional geometry: Implications for estimating body mass and physique from the skeleton
Source: Am J Phys Anthropol. 2018 Jan 18;166(1):56–69. doi: 10.1002/ajpa.23398 (PMC6178563; doi:10.1002/ajpa.23398)
Supplement: Supplementary file 1 — Supporting Information [file AJPA-166-56-s001.docx]

**Supplementary Table 1. Summary statistics for age, body size and body composition by group.** SD = standard deviation; Max. = maximum; Min. = minimum.

| **Variable** | **Rowers (n = 40)** | | | | **Distance runners (n = 11)** | | | | **Soccer players (n = 11)** | | | |  |
| --- | --- | --- | --- | --- | --- | --- | --- | --- | --- | --- | --- | --- | --- |
|  | **Mean** | **SD** | **Max.** | **Min.** | **Mean** | **SD** | **Max.** | **Min.** | **Mean** | **SD** | **Max.** | **Min.** | |
| Age (years) | 22 | 2 | 28 | 19 | 27 | 4 | 31 | 20 | 23 | 4 | 27 | 19 | |
| Stature (cm) | 174.0 | 6.5 | 188.2 | 163.3 | 169.1 | 8.0 | 184.0 | 160.0 | 164.4 | 4.4 | 169.9 | 155.8 | |
| Body mass (kg) | 69.3 | 9.0 | 90.0 | 51.8 | 56.2 | 7.6 | 70.6 | 45.4 | 63.6 | 5.7 | 74.3 | 56.7 | |
| Body mass index (kg/m^2^) | 22.8 | 2.2 | 30.6 | 18.3 | 19.6 | 1.9 | 23.1 | 15.6 | 23.5 | 2.0 | 28.0 | 21.3 | |
| Lean mass (kg) | 54.7 | 5.8 | 65.4 | 43.1 | 45.0 | 5.5 | 56.7 | 37.5 | 47.9 | 3.0 | 53.0 | 43.7 | |
| Fat mass (kg) | 14.2 | 4.8 | 30.9 | 5.4 | 12.0 | 3.2 | 19.6 | 8.5 | 15.6 | 4.5 | 25.6 | 9.9 | |
| % Fat mass (% of body mass) | 20.2 | 4.8 | 34.3 | 8.8 | 21.0 | 4.4 | 30.0 | 16.0 | 24.3 | 5.0 | 34.5 | 16.8 | |

*Supplementary table 1 cont.*

| **Variable** | **Ex-athlete**  **(gymnast: n = 1)** | **Ultrarunners (n = 8)** | | | | **Controls (n = 34)** | | | |
| --- | --- | --- | --- | --- | --- | --- | --- | --- | --- |
|  |  | **Mean** | **SD** | **Max.** | **Min.** | **Mean** | **SD** | **Max.** | **Min.** |
| Age (years) | 21 | 36 | 6 | 43 | 27 | 23 | 3 | 32 | 19 |
| Stature (cm) | 165.3 | 164.1 | 6.9 | 171.9 | 152.9 | 167.9 | 7.4 | 183.4 | 154.2 |
| Body mass (kg) | 51.5 | 60.1 | 6.9 | 72.4 | 52.7 | 61.7 | 11.1 | 92.1 | 40.0 |
| Body mass index (kg/m^2^) | 18.8 | 22.3 | 1.3 | 24.7 | 20.8 | 21.9 | 3.9 | 33.3 | 16.8 |
| Lean mass (kg) | 44.6 | 46.8 | 5.9 | 54.5 | 40.1 | 45.6 | 5.8 | 61.6 | 30.9 |
| Fat mass (kg) | 6.9 | 13.3 | 2.9 | 18.6 | 10.6 | 16.0 | 6.9 | 37.8 | 7.7 |
| % Fat mass (% of body mass) | 13.4 | 22.1 | 4.1 | 30.0 | 18.2 | 25.2 | 6.4 | 41.0 | 14.1 |

**Supplementary Table 2: Comparison of correlations between bone cross-sectional properties and body, lean and fat mass excluding rowers from the sample (“restricted sample”) with correlations for the full sample.** All variables were log transformed. ^a^ denotes statistically non-significant correlations (p > 0.05). TA = total area; CA = cortical area; MA = medullary area; *J* = polar second moment of area; *Zp* = polar section modulus.

|  | **Pearson correlation (restricted sample)** | | | | | | **Difference in correlation (full-restricted sample)** | | | | | |
| --- | --- | --- | --- | --- | --- | --- | --- | --- | --- | --- | --- | --- |
|  | **Unadjusted** | | | **Adjusted for stature** | | | **Unadjusted** | | | **Adjusted for stature** | | |
|  | **Body mass** | **Lean mass** | **Fat mass** | **Body mass** | **Lean mass** | **Fat mass** | **Body mass** | **Lean mass** | **Fat mass** | **Body mass** | **Lean mass** | **Fat mass** |
| **Humerus 50%** |  |  |  |  |  |  |  |  |  |  |  |  |
| TA (mm^2^) | 0.35 | 0.63 | -0.03 ^a^ | 0.19 ^a^ | 0.46 | -0.06 ^a^ | 0.15 | 0.05 | 0.13 | 0.06 | 0.08 | 0.06 |
| CA (mm^2^) | 0.38 | 0.67 | -0.03 ^a^ | 0.28 | 0.63 | -0.04 ^a^ | 0.16 | 0.06 | 0.06 | -0.05 | -0.03 | -0.10 |
| MA (mm^2^) | 0.22 ^a^ | 0.39 | 0.00 ^a^ | 0.05 ^a^ | 0.14 ^a^ | -0.03 ^a^ | 0.06 | -0.01 | 0.14 | 0.10 | 0.02 | 0.18 |
| *J* (mm^4^) | 0.38 | 0.67 | -0.03 ^a^ | 0.22 ^a^ | 0.52 | -0.05 ^a^ | 0.16 | 0.05 | 0.12 | 0.03 | 0.06 | 0.02 |
| *Zp* (mm^3^) | 0.37 | 0.65 | -0.02 ^a^ | 0.23 ^a^ | 0.54 | -0.05 ^a^ | 0.16 | 0.06 | 0.10 | 0.02 | 0.05 | -0.01 |
| Circumference (mm) | 0.36 | 0.65 | -0.03 ^a^ | 0.19 ^a^ | 0.46 | -0.05 ^a^ | 0.15 | 0.05 | 0.12 | 0.04 | 0.06 | 0.07 |
| Maximum diameter (mm) | 0.35 | 0.59 | -0.01 ^a^ | 0.18 ^a^ | 0.36 | -0.01 ^a^ | 0.07 | 0.00 | 0.08 | 0.07 | 0.18 | 0.00 |
| Minimum diameter (mm) | 0.32 | 0.54 | 0.02 ^a^ | 0.21 ^a^ | 0.46 | -0.03 ^a^ | 0.17 | 0.12 | 0.09 | 0.07 | 0.00 | 0.13 |
| **Femur 50%** |  |  |  |  |  |  |  |  |  |  |  |  |
| TA (mm^2^) | 0.46 | 0.74 | 0.07 ^a^ | 0.30 | 0.55 | 0.09 ^a^ | 0.12 | -0.01 | 0.13 | 0.02 | -0.10 | 0.10 |
| CA (mm^2^) | 0.47 | 0.71 | 0.10 ^a^ | 0.35 | 0.62 | 0.11 ^a^ | 0.08 | -0.03 | 0.08 | -0.01 | -0.13 | 0.06 |
| MA (mm^2^) | 0.15 ^a^ | 0.32 | -0.02 ^a^ | -0.03 ^a^ | -0.03 ^a^ | 0.00 ^a^ | 0.17 | 0.06 | 0.10 | 0.07 | 0.04 | 0.06 |
| *J* (mm^4^) | 0.46 | 0.74 | 0.07 ^a^ | 0.30 | 0.56 | 0.09 ^a^ | 0.11 | -0.02 | 0.12 | 0.00 | -0.11 | 0.10 |
| *Zp* (mm^3^) | 0.12 ^a^ | 0.35 | -0.11 ^a^ | -0.08 ^a^ | 0.03 ^a^ | -0.14 ^a^ | 0.22 | 0.17 | 0.09 | 0.07 | 0.15 | 0.05 |
| Circumference (mm) | 0.51 | 0.71 | 0.17 ^a^ | 0.39 | 0.56 | 0.20 ^a^ | 0.07 | -0.04 | 0.09 | -0.03 | -0.14 | 0.08 |
| Maximum diameter (mm) | 0.36 | 0.62 | 0.03 ^a^ | 0.19 ^a^ | 0.39 | 0.04 ^a^ | 0.22 | 0.09 | 0.18 | 0.16 | 0.07 | 0.19 |
| Minimum diameter (mm) | 0.48 | 0.73 | 0.10 ^a^ | 0.35 | 0.55 | 0.14 ^a^ | -0.02 | -0.13 | 0.06 | -0.16 | -0.27 | 0.00 |
| **Tibia 50%** |  |  |  |  |  |  |  |  |  |  |  |  |
| TA (mm^2^) | 0.50 | 0.69 | 0.20 ^a^ | 0.33 | 0.49 | 0.19 ^a^ | 0.11 | 0.03 | 0.07 | 0.06 | 0.02 | 0.09 |
| CA (mm^2^) | 0.48 | 0.63 | 0.20 ^a^ | 0.37 | 0.56 | 0.19 ^a^ | 0.08 | 0.02 | 0.04 | 0.00 | -0.07 | 0.04 |
| MA (mm^2^) | 0.22 ^a^ | 0.33 | 0.09 ^a^ | -0.01 ^a^ | -0.06 ^a^ | 0.06 ^a^ | 0.17 | 0.10 | 0.08 | 0.18 | 0.21 | 0.10 |
| *J* (mm^4^) | 0.49 | 0.68 | 0.19 ^a^ | 0.32 | 0.51 | 0.17 ^a^ | 0.11 | 0.03 | 0.06 | 0.06 | 0.01 | 0.10 |
| *Zp* (mm^3^) | 0.51 | 0.70 | 0.21 ^a^ | 0.36 | 0.51 | 0.21 ^a^ | 0.08 | 0.01 | 0.05 | 0.03 | -0.01 | 0.07 |
| Circumference (mm) | 0.49 | 0.67 | 0.21 ^a^ | 0.31 | 0.46 | 0.19 ^a^ | 0.11 | 0.02 | 0.07 | 0.08 | 0.02 | 0.11 |
| Maximum diameter (mm) | 0.37 | 0.57 | 0.11 ^a^ | 0.20 ^a^ | 0.41 | 0.04 ^a^ | 0.14 | 0.03 | 0.14 | 0.11 | -0.05 | 0.21 |
| Minimum diameter (mm) | 0.39 | 0.59 | 0.14 ^a^ | 0.21 ^a^ | 0.33 | 0.14 ^a^ | 0.12 | 0.04 | 0.04 | 0.11 | 0.11 | 0.03 |
